# Levetiracetam, lamotrigine and carbamazepine: which monotherapy during pregnancy?

**DOI:** 10.1007/s10072-021-05542-2

**Published:** 2021-09-01

**Authors:** Luisa Mari, Fabio Placidi, Andrea Romigi, Mario Tombini, Chiara Del Bianco, Martina Ulivi, Claudio Liguori, Natalia Manfredi, Alessandro Castelli, Nicola Biagio Mercuri, Francesca Izzi

**Affiliations:** 1 Epilepsy Centre, Department of System Medicine, Policlinico Tor Vergata, University of Rome Tor Vergata, Viale Oxford 81, 00133 Rome, Italy; 2 IRCCS Neuromed Istituto Neurologico Mediterraneo, Sleep Medicine Center, Via Atinense 18 , Pozzilli, IS Italy; 3 Unit of Neurology, Neurophysiology, Neurobiology, Department of Medicine, University Campus Bio-Medico, via Álvaro del Portillo 21, 00128 Rome, Italy

**Keywords:** Pregnancy, Epilepsy, Anti-seizure medications (ASMs), Monotherapy, Levetiracetam (LEV), Lamotrigine (LTG), Carbamazepine (CBZ)

## Abstract

**Objective:**

Epilepsy treatment during pregnancy is still challenging. The study is aimed at comparing the efficacy and safety of carbamazepine (CBZ), lamotrigine (LTG) and levetiracetam (LEV) monotherapies during pregnancy in women with focal (FE) or generalized (GE) epilepsy.

**Methods:**

A multicentre retrospective study was conducted to evaluate seizures frequency and seizure freedom (SF) rate during 3 months before pregnancy, each trimester of gestation and post-partum period in women on monotherapy with CBZ, LTG and LEV.

**Results:**

Fifty-seven pregnancies (45 FE, 12 GE) on monotherapy (29 CBZ, 11 LTG, 17 LEV) were included. A significant reduction of seizure frequency was found in the first trimester of pregnancy as compared with that one before pregnancy (*p* = 0.004), more evident in GE (*p* = 0.003) and in LEV group (*p* = 0.004). The SF rate significantly increased in the first trimester in comparison to that one before pregnancy and persisted in the post-partum period in the whole sample (*p* < 0.001) and in women on LEV (*p* = 0.004). Besides, 88.57% of SF women before pregnancy remained unchanged during gestation and the post-partum period. One major heart malformation in CBZ and no major malformations in LTG and LEV groups were found.

**Conclusions:**

A better clinical outcome during pregnancy emerged since the first trimester in comparison to the before-pregnancy period, mostly evident in women with GE and LEV therapy, reinforcing the hypothesis of a protective role of pregnancy versus seizures. SF before pregnancy represents a significant predictive factor of good clinical outcome during gestation and the post-partum period. Compared to CBZ, LTG and LEV showed a better safety profile.

## Introduction

Epilepsy in women is among the most critical issues in epileptology, since both epilepsy itself and anti-seizure medications (ASMs) may have several interactions with hormonal and reproductive systems and oral contraceptives in women of childbearing age [1]. A challenging issue is the treatment of epilepsy in women during pregnancy, whose prevalence is estimated between 0.3 and 0.5% [2].

According to data from the European Pregnancy Registry (EURAP), seizure frequency during gestation increases in 17.3%, decreases in 15.9% and remains unchanged in 63.6% of cases [3].

Furthermore, literature data about the course of epilepsy during pregnancy suggest that generalized epilepsy (GE) has a more favourable outcome than focal epilepsy (FE) [3, 4]. Moreover, although some authors report an increased risk of seizures in peripartum and post-partum periods in both groups [5, 6], according to EURAP, seizures occur during childbirth in less than 3% of pregnancies, which led the National Institute For Health and Care Excellence and the American Academy of Neurology (AAN) not to consider epilepsy as an indication for a caesarean section; therefore, spontaneous delivery is recommend, unless there is a high seizure frequency during pregnancy [5, 7]. Further studies report seizure control before pregnancy as an important predictive factor in determining good outcome of seizures during pregnancy [4, 5].

The problematic management of epilepsy during pregnancy is also related to ASMs’ exposition associated with an increased risk of major congenital malformations (MCMs), defined as anatomical/structural/functional, or purely aesthetic, abnormalities requiring corrective surgery [8]. As in healthy subjects not exposed to ASMs, MCMs mainly occur in the first trimester between the eighth and the tenth week of gestation and appear to be associated with folic acid deficiency, whose supplementation can reduce risk between 60 and 86% [9]. According to an extensive meta-analysis, the risk of MCMs in pregnancy in untreated epileptic women appears similar to that of the general population or 1.92% (OR = 1.92; 95% CI 0.92–4.00) [10]. In the last two decades, several international registers, such as the EURAP, the North American Antiepileptic Drug and Pregnancy Registry (NAAPR) and the English-Irish registry (UK and Ireland Pregnancy Register), showed that the highest prevalence of MCMs had been associated with valproic acid, whereas lower prevalences have been associated with lamotrigine (LTG) and levetiracetam (LEV); drugs such as phenobarbital, topiramate, phenytoin and carbamazepine (CBZ) confer an intermediate risk of congenital malformations [11]. Data on new ASMs, such as lacosamide, brivaracetam, eslicarbazepine and perampanel, are still lacking [12]. Consistently with the above scenario, a notable increase in the use of LTG and LEV and a parallel decrease in valproic acid and CBZ were reported by the UK and Ireland Pregnancy Registry [13].

Such evidence suggests that epilepsy management in pregnant women requires avoiding polytherapy, especially when it includes VPA, and the prescription of the lowest effective dose for reaching an optimal level in the control of epileptic seizures with minimal adverse effects on the foetus [14].

In our retrospective study, the primary objective was to assess the clinical outcome evaluating seizure frequency and seizure freedom (SF) rate during the three trimesters of pregnancy and in the post-partum period in women affected by FE or GE on monotherapy with CBZ, LTG or LEV.

## Methods

We conducted a multicentre retrospective study on pregnant women with FE or GE followed at the Epilepsy Center of Policlinico Tor Vergata and the Epilepsy Center of Policlinico Campus Bio-Medico in the period between 2009 and 2019, including women on ASMs with CBZ, LTG or LEV.

For each patient, the following data were recorded: age at the time of pregnancy, epilepsy type (FE or GE) and its aetiology (genetic, structural or unknown epilepsy) and seizure type (i.e. generalized tonic–clonic seizures, generalized motor seizures, generalized absences seizures, focal aware seizure, focal impaired awareness seizure and focal to bilateral tonic–clonic seizure) according to the International League Against Epilepsy (ILAE) classification [15–17]. We also collected data on the daily dose of ASMs monotherapy at the beginning of pregnancy, and any changes in ASMs carried out during gestation.

On the basis of the seizures occurrence before pregnancy (3 months before pregnancy), patients were classified as seizure free (no seizures), with sporadic frequency (less than one seizure per month), monthly, weekly and daily frequency.

During each trimester of pregnancy and in the post-partum period, defined as 3 months after childbirth, women underwent periodic clinical follow-up visits through specialist medical examination and foetal screening tests.

A subgroup of women underwent standard 20-min video-EEG recordings before the pregnancy period, during each trimester of pregnancy and in the post-partum period. We also investigated any pathologies or complications during pregnancy, any additional required therapies, foetal malformations found at screening tests and the type of birth conducted (caesarean section or natural).

Seizures occurrence for each trimester has been reported by women into a clinical diary; thus, the change in the number of seizures and SF rate has been compared between (i) before pregnancy (3 months before pregnancy), (ii) I trimester, (iii) II trimester, (iv) III trimester of pregnancy and (v) post-partum (3 months following delivery).

The study was conducted according to regulations of the Independent Ethical Committee of the Policlinico Tor Vergata and Policlinico Campus Bio-Medico; an informed consent was obtained from each patient for the processing of personal data.

### Statistical analysis

Descriptive statistics are reported as absolute numbers, percentage and mean ± standard deviation. We carried out non-parametric tests after assessing non-normality distribution by Shapiro–Wilk and Kolmogorov–Smirnov normality tests for all variables; to evaluate numerical variables change over time within-group analysis, Friedman and Wilcoxon tests were applied. Between-groups analysis was performed through Kruskal–Wallis and Mann–Whitney tests. Pearson chi-square test was used to compare categorical variables (SF percentage) between different groups and Q Cochran test and McNemar tests for within-group analysis, to evaluate changes over time.

Statistical analyses were performed with 4 IBM SPSS Statistics 23 program. Statistical significance was set at *p* < 0.05; where appropriate, Bonferroni’s correction has been applied, setting *p* at < 0.0167 or at < 0.01 for 3 or 5 multiple comparisons, respectively.

## Results

### Demographical and clinical data

In this retrospective study, 57 pregnancies in 52 women with FE or GE undergoing ASMs monotherapy with CBZ, LTG or LEV were analysed. Forty-five women were followed at the Epilepsy Center of Policlinico Tor Vergata, and seven women were observed at the Epilepsy Center of Policlinico Campus Bio-Medico. Mean age at the time of pregnancy was 31.28 ± 5.25 years. Two pregnancies were considered for five women as they occurred in the observation period and were conformed to the inclusion criteria.

Of the 57 pregnancies, 45 were conducted by 41 women with FE including 3 with structural epilepsy; 5 women had focal without impairment seizures, 17 women had focal impaired awareness seizures and 19 women had focal to bilateral tonic clonic seizures. More in detail, 13 women had temporal focal epilepsy with seizures characterized by déjà-vu, psycho-motor arrest, automatisms, tinnitus and/or epigastric aura; one patient had seizures with speech arrest; and two women had only nocturnal seizures.

Twelve pregnancies were conducted by 11 women with GE (7 patients with generalized tonic–clonic seizures, 1 patient with juvenile myoclonic epilepsy and 3 women had epilepsy with eyelid myoclonus and absences).

All pregnancies in our study were conducted on ASMs monotherapy: 29 on CBZ, 11 on LTG and 17 on LEV; mean daily dose of each ASM and seizure frequency is reported in Table 1.

**Table 1:** Demographic and clinical data before pregnancy

	Total *n* = 57	FE *n* = 45	GE *n* = 12	CBZ *n* = 29	LTG *n* = 11	LEV *n* = 17
Age at pregnancy, y (mean ± SD)	31.28 ± 5.25	31.73 ± 7.77	29.58 ± 1.41	30.89 ± 5.4	34.45 ± 4.13	29.88 ± 5.04
Epilepsy type FE/GE	45/12	45/0	0/12	27/0	8/3	8/9
Seizure type		5FAS, 17FIAS, 19FTBCS	7GTCS,1GMS 3GAS	14 FTBCS, 12 FIAS, 3 FAS	2 GTCS, 1 GMS, 6 FIAS, 2 FTBCS	3GAS, 6 GTCS, 4 FTBCS, 2 FAS, 2 FIAS
Seizure frequency, *n*/trimester (mean ± SD)	2.65 ± 11.22	2.90 ± 12.53	1.75 ± 3.37	4.03 ± 15.57	1.90 ± 3.56	0.79 ± 1.07
Seizure free	35	29	6	19	6	10
Sporadic	9	5	4	2	2	5
Monthly	10	9	1	6	2	2
Weekly	2	1	1	1	1	0
Daily	1	1	0	1	0	0
ASM daily dose, mg (mean ± SD)	na	na	na	576.66 ± 277.5	168.18 ± 78.3	1250 ± 625.8

*FE* focal epilepsy, *GE* generalized epilepsy, *CBZ* carbamazepine, *LTG* lamotrigine, *LEV* levetiracetam, *FTBCS* focal to bilateral tonic–clonic seizure, *FIAS* focal impaired awareness seizure, *FAS* focal without impaired awareness seizure, *GTCS* generalized tonic–clonic seizure, *GMS* generalized motor seizures, *GAS* generalized absences seizure, *na*, not applicable, *ASM* anti-seizure medication

No statistical differences were observed in terms of age at pregnancy and SF rate before pregnancy, between FE and GE groups and between LTG, CBZ and LEV groups (*p* > 0.05). In particular, seizure frequency before pregnancy was comparable in FE and GE groups (*p* = 0.57) and between LTG, CBZ and LEV groups (*p* = 0.86).

Seizure frequency during pregnancy.

Considering the whole sample, a significant change in seizure frequency during the pregnancy was found (*p* = 0.004); the improvement was significant between the 3 months before pregnancy and the first trimester (2.65 ± 11.22 vs 2.08 ± 11.18, *p* = 0.004), while no significant variation was recorded between the three different trimesters and between the before-pregnancy period and the post-partum period.

Even considering the sample by subgroups based on epilepsy type, in the GE group, although there was a significant reduction in seizure frequency during pregnancy compared to the before-pregnancy period (*p* = 0.003), such finding loses statistical significance after Bonferroni’s correction; for the group with FE, on the other hand, no significant differences emerged in terms of seizure frequency in all of the periods considered (Table 2).

**Table 2:** Seizure frequency during pregnancy by epilepsy type

	TOT *n* = 57	FE *n* = 45	GE *n* = 12
Before pregnancy	2.65 ± 11.22	2.90 ± 12.53	1.75 ± 3.37
I Trimester	2.08 ± 11.18	2.53 ± 12.57	0.41 ± 0.90
II Trimester	3.60 ± 15.58	4.50 ± 17.46	0.25 ± 0.86
III Trimester	2.35 ± 11.34	2.92 ± 12.73	0.25 ± 0.86
Post-partum	4.07 ± 22.39	4.98 ± 25.17	0.62 ± 1.18
*p* value*	**0.004**	0.098	**0.003**
*p* value#	**0.004**	0.041	0.027
*p* value£	0.354	0.937	0.024
*p* valueϯ	0.075	0.445	0.024

Data are expressed as mean ± SD. *FE* focal epilepsy, *GE* generalized epilepsy. Statistical analysis: *, Friedman test; #, Wilcoxon between before pregnancy and I trimester; £, Wilcoxon between before pregnancy and II trimester; ϯ, Wilcoxon between before pregnancy and III trimester. Bold values denote statistical significance. After Bonferroni’s correction statistical significance value set at *p* < 0.01

As to the analysis by subgroups for monotherapy, no significant differences, in terms of seizure frequency in both the CBZ and LTG groups, were found, whereas a significant reduction in seizure frequency (*p* = 0.004) was observed in LEV group, in particular between before-pregnancy period and the first trimester (0.79 ± 1.07 vs 0.05 ± 0.24, *p* = 0.016) and between before-pregnancy period and the third trimester (0.79 ± 1.07 vs 0.05 ± 0.024, *p* = 0.016) closer to statistical significance (Table 3).

**Table 3:** Seizure frequency during pregnancy by ASM monotherapy

	CBZ *n* = 29	LTG *n* = 11	LEV *n* = 17
Before pregnancy	4.03 ± 15.56	1.90 ± 3.55	0.79 ± 1.07
I Trimester	3.77 ± 15.61	0.77 ± 1.21	0.05 ± 0.24
II Trimester	6.41 ± 21.53	1.50 ± 3.61	0.17 ± 0.72
III Trimester	4.03 ± 15.69	1.50 ± 3.61	0.05 ± 0.24
Post-partum	7.03 ± 31.28	1.63 ± 3.64	0.58 ± 1.04
*p* value*	0.741	0.246	**0.004**
*p* value#	0.258	0.066	0.016
*p* valueϯ	0.615	0.408	0.016

Data are expressed as mean ± SD. *CBZ* carbamazepine, *LTG* lamotrigine, *LEV* levetiracetam. Statistical analysis: *, Friedman test; #, Wilcoxon test between before pregnancy and I trimester; ϯ, Wilcoxon test between before pregnancy and III trimester. Bold values denote statistical significance. After Bonferroni’s correction statistical significance value set at *p* < 0.01

Seizure freedom rate during pregnancy.

Thirty-one of the 35 (88.57%) women SF before pregnancy remained SF during all pregnancy and post-partum period; moreover, considering the 22 pregnancies of non-SF women, 6 of them (4 with sporadic seizures and 2 with monthly seizures) became SF from the first trimester of pregnancy and remained SF for the entire duration of pregnancy and in the post-partum period, while two women with sporadic seizures became SF from the second trimester of pregnancy and remained such even in post-partum period; 3 women (2 with monthly seizure and 1 with sporadic seizures) became SF during pregnancy and had seizure recurrence in post-partum period.

Thus, a significant change in percentage of SF along pregnancy in the whole sample was observed (*p* < 0.001) consisting in an increase of SF percentage that was evident between before-pregnancy period and first trimester (61.4% vs 78.9%, *p* = 0.006) and to a lesser extent in post-partum period (61.4% vs 77.2%, *p* = 0.022) (Table 4).

**Table 4:** Seizure freedom rate during pregnancy by epilepsy type

	total *n* = 57	FE *n* = 45	GE *n* = 12
Before pregnancy *n* (%)	35 (61.4)	29 (64.44)	6 (50)
I Trimester *n* (%)	45 (78.9)	36 (80)	9 (75)
II Trimester *n* (%)	46 (80.7)	35 (77.77)	11 (91.66)
III Trimester *n* (%)	47 (82.5)	36 (80)	11 (91.66)
Post-partum *n* (%)	44 (77.2)	34 (75.55)	10 (83.3)
*p* value*	** < 0.001**	0.027	**0.010**
*p* value#	**0.006**	0.039	0.250
*p* value£	0.022	0.18	0.125

*FE* focal epilepsy, *GE* generalized epilepsy. Statistical analysis: *, Q Cochran test; #, McNemar between before pregnancy and I trimester; £, McNemar between before pregnancy and post-partum. Bold values denote statistical significance. After Bonferroni’s correction statistical significance value set at *p* < 0.01

Concerning the subgroup analysis, and based on epilepsy type, a statistically significant increase in the percentage of SF women in both groups (FE and GE) was observed on the Q Cochran test. However, such finding loses statistical significance at within-group analysis at McNemar test (Table 4).

Regarding the 45 pregnancies of FE women, 26 out of 29 SF before pregnancy remain SF during all pregnancy and in post-partum period, while of the 16 non-SF women, 4 became SF during gestation remaining SF in the post-partum period.

Only 2 SF women with FE experienced seizures during pregnancy; more in detail, one patient with FE had seizures since the first trimester with monthly frequency forcing the reintroduction of therapy with CBZ up to 600 mg/day (suspended before pregnancy), while the second patient had seizures with monthly frequency during the second trimester of pregnancy, maintaining unchanged her therapy (CBZ 600 mg/day). One patient remained SF during all pregnancy and had a recurrence of seizures in the post-partum period.

With special reference to the 12 pregnancies of women with GE, all 6 SF women before pregnancy remained unchanged during all gestation, while 5 of them remained SF also in the post-partum period. In addition, considering the 6 non-SF women before pregnancy, 5 of them (4 women with sporadic seizures, one patient with monthly seizures) became SF since I or II trimester of pregnancy; of such latter women, 4 remained SF in the post-partum period.

Taking into account the monotherapy subgroup analysis, no significant statistical changes in the number of SF women were observed, either in CBZ group or in LTG group, while a significant increase in SF women’s percentage during gestation was observed in LEV group (*p* = 0.004), in particular between the before-pregnancy period and the I trimester of pregnancy (58.82% vs 94.11% *p* = 0.031), even if losing statistical significance after Bonferroni’s correction (Table 5). Eight out of 10 SF women on LEV therapy before pregnancy remained SF both during gestation and post-partum period. Six out of 7 non-SF women became SF during pregnancy, and 4 of them remained SF also in the post-partum period.

**Table 5:** Seizure freedom rate during pregnancy by ASM monotherapy

	CBZ *n* = 29	LTG *n* = 11	LEV *n* = 17
Before pregnancy *n* (%)	19 (65.51)	6(54.54)	10 (58.82)
I Trimester *n* (%)	22(75.86)	7(63.63)	16 (94.11)
II Trimester *n* (%)	22 (75.86)	8(72.72)	16 (94.11)
III Trimester *n* (%)	23 (79.31)	8(72.72)	16 (94.11)
Post-partum *n* (%)	23 (79.31)	8 (72.72)	13 (76.47)
*p* value*	0.249	0.171	**0.004**
*p* value#	0.375	1	0.031
*p* value£	0.219	0.5	0.375

*CBZ* carbamazepine, *LTG* lamotrigine, *LEV* levetiracetam. Statistical analysis: *, Q Cochran test; #, McNemar between before pregnancy and I trimester; £, McNemar between before pregnancy and post-partum; *ns* not significant. Bold values denote statistical significance. After Bonferroni’s correction statistical significance value set at *p* < 0.01

Video-EEG monitoring during pregnancy.

Twenty-four women underwent video-EEG monitoring during the before-pregnancy period, in each trimester of pregnancy and during the post-partum period.

Before pregnancy, 17 women had interictal epileptiform discharges, six women had only no specific abnormalities and one patient had normal EEG. As to the 17 women with interictal epileptiform discharges, 8 (47%) showed an improvement of EEG pattern during pregnancy or in the post-partum period, whereas EEG pattern remained unchanged in 9 (53%) women. Only one patient with GE and not specific abnormalities before pregnancy showed an EEG pattern deterioration with the appearance of interictal epileptiform discharges in the first trimester of pregnancy and the post-partum period; however, no concomitant seizure worsening occurred, and no therapy changes were required.

Changes in ASMs therapy and supplemental drugs during pregnancy.

In 46 out of 57 pregnancies, ASMs therapy remained unchanged during gestation. Four women on CBZ therapy needed a drug dosage increase due to seizure worsening during pregnancy; one patient with FE on CBZ therapy (400 mg/day) slightly deteriorated from sporadic to monthly seizures concomitant with iatrogenic hepatitis in the second trimester, requiring a therapeutic shift to LEV (1500 mg/day). One FE patient, who had withdrawn CBZ treatment before pregnancy due to SF, had to be treated with CBZ 600 mg/day for seizure recurrence in her first trimester of gestation. With regard to the 11 pregnancies on LTG therapy, an increase in drug dosage was required only in 4 women with FE (in 3 for seizure worsening and in the remaining one for plasma LTG levels reduction). None of the 13 pregnancies on LEV therapy needed changes to the daily dosage.

Forty-eight out of fifty-seven women (84%) underwent a folic acid supplementation therapy at a dosage of 5 mg/day; 7 women have also taken iron supplementation for the development of gestational anaemia, 2 women were treated with levothyroxine for hypothyroidism, one patient with progesterone, 1 with antispasmodics, 1 with antibiotic therapy, 6 with antiplatelet, 5 with anticoagulant therapy and 2 with supplementation of vitamin K.

Complications during pregnancy.

None of the 57 pregnancies developed status epilepticus during gestation. No maternal death or abortion occurred. Nine complications were observed in our sample: 2 women, on CBZ and LEV therapy respectively, developed anaemia; 2 CBZ women had gestosis; 2 women on CBZ had oligohydramnios/polyhydramnios; one CBZ patient had iatrogenic hepatitis; one LTG patient developed gestational diabetes.

All women underwent to morphological ultrasound, 35 to foetal echocardiography, 26 to bi-test and 14 to amniocentesis. Instrumental monitorings showed only one major foetal malformation: one patient on CBZ therapy gave birth to a stillborn foetus because of bilateral renal agenesis; however, the same patient had another complication-free pregnancy on CBZ therapy. Four minor congenital malformations emerged: two interventricular septal defects and one foramen ovale on CBZ therapy and one renal pyelectasis on LEV therapy.

Childbirth.

**Fig. 1:**
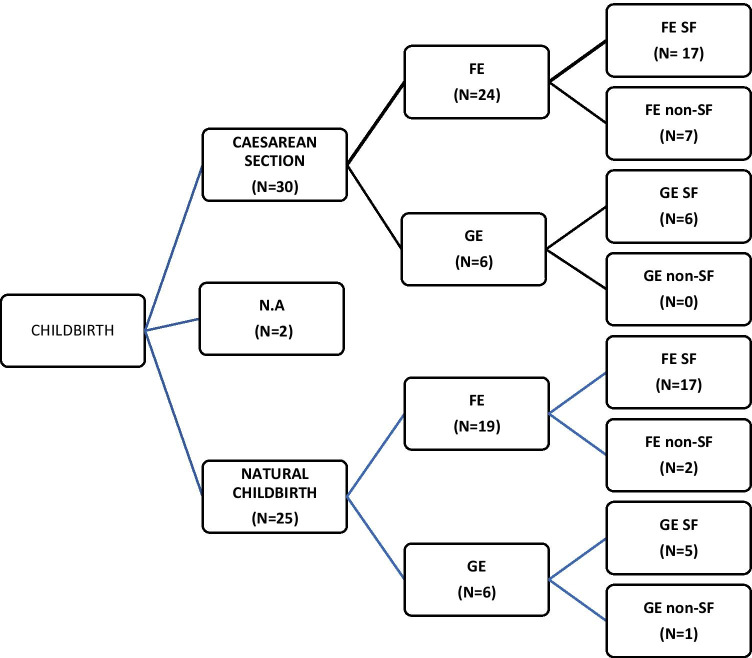
Childbirth: caesarean section and natural childbirth by epilepsy type in seizure free (SF) and in non-SF patients. Abbreviations: FE focal epilepsy, GE generalized epilepsy, SF seizure freedom, N.A. not available

Pregnancies ended with 30 (52.63%) caesarean section, 25 (43.86%) natural birth and 2 (3.51%) unknown outcome. Considering the 45 pregnancies of women with FE, 36 were SF in the third trimester, 17 underwent caesarean section and 17 natural delivery. Of the 12 GE pregnancies, 11 were SF in the third trimester; 6 underwent caesarean section and five natural delivery (Fig. 1).

## Discussion

Epilepsy treatment in pregnant women is challenging, requiring an optimal control of seizures while guaranteeing minimal adverse effects on the foetus [11]. The peculiarity of this study was to compare the efficacy and safety of ASMs in a targeted selection of FE and GE women on monotherapy with CBZ, LTG and LEV, evaluating seizure control through a systematic analysis in each of three trimesters of pregnancy and the post-partum period.

The study, based on the clinical outcome of seizures during pregnancy, showed a significant increase of SF rate in the first trimester of gestation (*p* = 0.006), which persisted in a lesser extent in the post-partum period (*p* = 0.022); in addition, a significant reduction of the seizure frequency during the whole gestation period was observed (*p* = 0.004), with a notable improvement already from the first trimester (*p* = 0.004).

Moreover, most of the women SF in the three months before gestation showed a tendency to remain unchanged during pregnancy and in the post-partum period without any therapeutic changes. This finding is consistent with studies reporting that seizure control before pregnancy is an important predictive factor in determining good clinical outcome during pregnancy [4, 5]; more in detail, women on ASMs which were SF 9 months before pregnancy exhibited a probability between 84 and 92% of remaining SF, even during gestation, keeping their usual therapeutic regimen [5].

Taking into account possible effects of pregnancy on epilepsy, literature data are discordant. The Australian registry reported that women with epilepsy, even those not treated with ASMs, have a higher frequency of seizures during gestation compared to the before-pregnancy year, thus suggesting a possible worsening role of pregnancy itself on seizures [18]. The explanation can be related to the augmentation of plasma volume and drug metabolism, resulting in a reduction in the ASMs plasma levels [19], and to conditions such as psychological stress and sleep deprivation, which are not uncommon in pregnancy and may lead to a reduction of the epileptogenic threshold. However, some authors observed that seizure frequency during pregnancy does not vary in 53.2–63.6% of cases and is reduced in 15.9–22.7% of cases [3, 20]. Such latter findings appear to be consistent with our data; indeed only in 7 pregnancies (6 FE and 1 GE), we observed a clinical worsening, reinforcing the hypothesis of a protective role of pregnancy in epilepsy. Indeed, experimental models suggest a protective role of progesterone (whose levels rise during pregnancy) in determining an increase of the epileptogenic threshold [21].

Concerning the role of the epilepsy type on clinical outcome during pregnancy, both GE and FE women of our sample showed an increase of the SF rate during pregnancy compared to the before-pregnancy period, although without statistical significance; only the women with GE showed a significant reduction in seizure frequency during gestation compared to the before-pregnancy period, although this data loses again statistical significance after Bonferroni’s correction probably because of the small sample size. A better clinical outcome during gestation in GE, in comparison to FE, has been observed [3]; the two different types of epilepsy seem to show a dissimilar trend, with seizure recurrence during the first and in the third trimester of pregnancy in FE, and a single peak of relapse in the first trimester, as may occur in GE [3, 4]. The better prognosis in women with GE may be traced in few experimental studies on murine models; indeed progesterone, acting on receptors located at substantia nigra and basal ganglia levels, may prevent generalization of the seizures [22]. In addition, more recent studies showed how the progesterone inhibitory role on seizures is both dose- and time-dependent, with lower doses needed and longer effect duration in controlling generalized seizures, as compared to focal ones [21].

Furthermore, the comparison analysis among three different monotherapies showed a statistically significant increase in the SF rate (*p* = 0.004), together with a significant reduction in seizure frequency (*p* = 0.004) during pregnancy only in women treated with LEV, mostly occurring between the before-pregnancy period and the first trimester of gestation. On the contrary, we did not find any statistically significant seizure frequency changes during pregnancy, both in CBZ and LTG groups.

Considering the efficacy of the various ASMs during pregnancy, it has been reported that the risk of seizures in women on monotherapy is similar for LEV and CBZ (31.8% and 37.8%, respectively) and higher for LTG (51.3%) [23], while other authors reported that LEV is more effective than LTG, especially in women with GE [24]. Our data partially confirm previous findings, since we did not find differences between CBZ and LTG on seizure control, while it is of noteworthy that our women on LEV monotherapy showed a better outcome during gestation, irrespective of the type of epilepsy as half of them was affected by FE.

Variable efficacy of several ASMs during pregnancy has been previously related to blood levels changes [19]. In our study, LEV, LTG and CBZ blood levels were not routinely performed in all women, as the clinical outcome drove the therapeutic management. According to literature data, CBZ blood levels remain steady during pregnancy making unnecessary to monitor them [25]; on the contrary, both LEV and LTG were subject to metabolism changes during pregnancy, and therefore, EURAP recommends their monthly monitoring in order to adjust drug dosages and to ensure efficacy on seizures control. Oestrogens are known to increase up to 200% the renal clearance of LTG, especially in the third trimester [26]; thus causing the drug to be less effective during pregnancy. Consistently with these findings, EURAP reports an average LTG dose increase of 26%, and it has been shown that, following a close monitoring set-up, the risk of increased seizure frequency is not higher than other ASMs [27]. Also, an increase of 40–60% in metabolism for LEV may occur [28] in the third [29, 30] or the first [31] trimester of gestation, although, unlike the LTG, the blood LEV reduction requires dosage increases only in 15.9% of case [23]. In our sample, an increase of drug dosage was carried out due to poor seizure control in 36.6% of women on LTG, in 13.79% of women on CBZ and in no patient on LEV monotherapy. Only one patient on LTG had to increase the drug dosage due to reduced blood levels. Hence, our data suggest that in clinical practice, therapeutic adjustments during gestation are only rarely necessary and that the monitoring of drug serum levels is not always required [32], while clinical outcome may efficiently drive ASMs changes. Indeed, it is still unclear whether the blood reduction of LEV and LTG levels has implications in terms of seizure recurrence risk or increased seizure frequency [28, 30, 32–34].

As to the additional therapies, 84% of our women were administered with folic acid at the dosage of 5 mg/day according to AAN guidelines, which recommend folic acid supplementation from 0.4 to 5 mg/day [5] to reduce risks of neural tube defects of about 60–86% [9, 35] and spontaneous abortion of about 14.5–5.7% [36]. No abortions occurred in our sample, and only one major renal congenital malformation in a child exposed to CBZ was found. We observed minor congenital heart malformations in three foetuses exposed to CBZ, a minor kidney defect in one foetus exposed to LEV and no malformations in foetuses exposed to LTG. Our data are in line with those of several international registers (EURAP, NAAPR and UK and Ireland) which reported a lower malformation risk for LTG and LEV (1.9–2.9% and 0.7–2.8%, respectively) and an intermediate dose-dependent risk for CBZ (2.6–5.6%).

With regard to the type of delivery, 54.5% of our sample completed the pregnancy with caesarean section, despite a high percentage of women (76.6%) being SF in the last trimester of pregnancy. This finding conflicts with EURAP recommendations which suggest not to consider epilepsy an indication for caesarean section, unless there is a high seizure frequency during pregnancy, as the seizure recurrence risk during childbirth is only 3%. So far, the reason for the higher caesarean section rate in women with epilepsy has not been sufficiently investigated. We hypothesize that it comes from the “fear” that labour may provoke seizures rather than from strictly obstetric-gynaecological requirements [37]. However, our data are similar to previous studies that reported an incidence of caesarean section in women with epilepsy ranging from 66.7 to 85.33% [38, 39].

We are aware of the limits of our study. Firstly, it was conducted with a retrospective design, although such method represents the standard practice in women with epilepsy observation during pregnancy due to ethical reasons. Secondly, in our sample, FE is more represented than GE; however, this is consistent with the incidence and prevalence distribution of the two types of epilepsy. Thirdly, ASMs levels before and during pregnancy were not available in all patients, even if their clinical implication is still unclear.

In conclusion, our study shows a better clinical outcome of seizures during pregnancy since the first trimester in comparison to the before-pregnancy period, more evident for GE and for women on LEV monotherapy, reinforcing the hypothesis of a protective role of pregnancy versus epilepsy. Our findings confirm the well-known safety data on CBZ, LTG and LEV monotherapies during pregnancy with a better profile for LTG and LEV; in addition SF before pregnancy represents a significant predictive factor of good clinical outcome during gestation, as well in the post-partum period. Finally, our data together with previous reports [40] suggest that therapeutic adjustments during pregnancy can be driven by clinical course and that monitoring of drug serum levels is rarely necessary.
